# Sustainable Gamma-Crosslinked Hyaluronic Acid-Carbon Quantum Dots Films for Active Packaging and Extended Fruit Shelf Life

**DOI:** 10.3390/nano16181129

**Published:** 2026-09-10

**Authors:** Abdulhakeem Alzahrani, Suleiman A. Althawab

**Affiliations:** Department of Food Science & Nutrition, College of Food and Agricultural Sciences, King Saud University, Riyadh 11451, Saudi Arabia

**Keywords:** hydrogel, carbon dots, antioxidant, biocompatibility, food packaging

## Abstract

This study presents a sustainable approach for developing multifunctional hyaluronic acid–TMSPMA hydrogel films incorporated with banana stem-derived carbon dots (BCDs) via gamma radiation. The solvent-free radiation process enables sterile synthesis, homogeneous crosslinking, and uniform BCD distribution. FTIR and XPS confirmed oxygen- and nitrogen-rich functional groups on BCDs; TGA demonstrated excellent thermal stability. The nanocomposite films (HT series) exhibited improved structural integrity, enhanced thermal resistance, and smooth compact morphology. Cytocompatibility verified non-toxicity and biocompatibility. Surface and barrier analyses indicated increased hydrophilicity and reduced oxygen/moisture permeability. The films showed strong antioxidant performance exceeding 60% (DPPH) and 70% (ABTS) radical scavenging for HT3, and a controlled, diffusion-mediated BCD release. In banana packaging, the films delayed ethylene and CO_2_ evolution, prolonging shelf life and reducing greenhouse gas emissions. Overall, this work introduces a green and scalable route for producing BCD-reinforced hydrogel films combining antioxidant functionality, biocompatibility, and biodegradability for next-generation active food packaging.

## 1. Introduction

The growing environmental crisis caused by non-biodegradable plastic waste has accelerated the search for sustainable alternatives in food packaging [1]. Conventional petroleum-based polymers, while effective in preserving food quality, contribute significantly to global plastic pollution, microplastic contamination, and greenhouse gas emissions [2]. In response, biodegradable and biocompatible materials derived from natural sources have gained increasing attention as substitutes for single-use plastics. Among them, polysaccharide- and protein-based hydrogels have shown remarkable promise due to their inherent biodegradability, mechanical tunability, and ability to incorporate functional nanomaterials [3]. The integration of nanotechnology into biopolymer matrices has further enabled the design of “active” packaging systems capable of extending food shelf life, monitoring freshness, and preventing microbial contamination [4].

Carbon dots (CDs), a novel class of carbon-based nanomaterials, have emerged as sustainable functional fillers for developing next-generation packaging materials [5,6,7]. CDs possess unique optical, electronic, and chemical properties, including tunable fluorescence, high water dispersibility, strong antioxidant activity, and excellent biocompatibility [8]. Their abundant surface functional groups allow easy modification and strong interactions with polymeric matrices, leading to improved mechanical stability, barrier performance, and antimicrobial efficacy [9]. In recent years, the synthesis of CDs from biomass waste such as fruit peels, agricultural residues, and plant stems has gained momentum as a green and cost-effective route toward carbon nanomaterials [10,11,12]. Such bio-derived CDs not only reduce environmental waste but also align with circular economy principles by converting low-value biomass into high-value nanostructures [13]. Banana stem, a largely discarded agricultural byproduct, represents an abundant and renewable biomass source rich in cellulose, hemicellulose, and lignin ideal precursors for carbonization. The conversion of banana stem into CDs provides a dual environmental benefit: waste valorization and sustainable nanomaterial production [14]. Recent studies have demonstrated the use of banana-derived CDs for fluorescence sensing, antioxidant activity, and biomedical applications; however, their potential for integration into biopolymer-based food packaging films remains underexplored [15]. When incorporated into a hydrogel network, such CDs can impart antioxidant and antimicrobial properties, which are crucial for maintaining food quality and extending shelf life [16]. Moreover, their surface chemistry allows for strong interactions with hydrophilic polymers, improving film uniformity, transparency, and stability.

Among various biodegradable polymers, hyaluronic acid (HA) has gained attention due to its excellent biocompatibility, moisture retention ability, and film-forming capacity [17]. As a natural polysaccharide, HA plays a critical role in biological systems and is recognized as safe for food and biomedical use [18,19,20]. However, HA-based hydrogels often exhibit limited mechanical strength and barrier properties, restricting their direct application in packaging. To overcome these challenges, functional crosslinkers and silane-based coupling agents have been introduced to enhance the structural integrity of HA films. One such coupling agent, 3-(Trimethoxysilyl)propyl methacrylate (TMSPMA), can covalently link organic and inorganic components, creating a reinforced network that enhances film stability without compromising biodegradability or safety [21].

Gamma irradiation offers a clean and efficient method for hydrogel synthesis and polymer crosslinking without the need for additional chemical initiators or catalysts [22]. It induces polymerization and grafting reactions through high-energy ionization, resulting in a uniform network structure [23]. Importantly, gamma irradiation avoids the generation of toxic residues and ensures sterilization, making it particularly attractive for food-related applications [24]. Recent research has demonstrated that radiation-assisted fabrication can improve the homogeneity, transparency, and functional performance of nanocomposite hydrogels [25]. When combined with natural polymers and nanofillers, this technique enables the production of films with superior mechanical, barrier, and bioactive properties [26]. The consolidation of banana stem-derived carbon dots within a gamma-irradiated hyaluronic acid–TMSPMA hydrogel framework thus represents a promising strategy for developing multifunctional, sustainable, and biocompatible food packaging materials [27]. This approach synergistically combines the renewable origin and bioactivity of biomass-derived CDs with the film-forming ability and biocompatibility of HA, while TMSPMA provides the necessary chemical linkage and network reinforcement. Such a system not only addresses environmental and health concerns but also offers enhanced spoilage [28]. Furthermore, the use of gamma radiation eliminates the need for chemical crosslinkers and enhances the safety profile of the resulting films [29]. Recent advancements worldwide have highlighted the growing interest in active and intelligent packaging systems [30]. Researchers are developing films that can detect spoilage gases, regulate oxygen permeability, or release antimicrobial agents on demand [31]. Biopolymer–nanocomposite films incorporating carbon nanomaterials, metal oxides, and natural extracts are being explored for their ability to enhance preservation and provide real-time freshness indicators. However, challenges persist in achieving scalable, cost-effective, and non-toxic fabrication routes that maintain biocompatibility and meet food safety standards [32]. In this context, the current work aligns with global efforts toward sustainable nanotechnology and circular bioeconomy by utilizing agricultural waste, green synthesis, and radiation-assisted fabrication methods.

This study reports, for the first time, a gamma radiation-assisted synthesis of hyaluronic acid–TMSPMA hydrogel films embedded with banana stem-derived carbon dots for biocompatible active food packaging. Unlike conventional chemical or thermal fabrication routes, the use of gamma radiation ensures a solvent-free, initiator-free, and sterile synthesis process. The incorporation of bio-derived CDs enhances antioxidant, mechanical, and barrier properties while maintaining non-cytotoxicity and biodegradability. The developed nanocomposite film demonstrates the feasibility of integrating sustainable carbon nanomaterials with biopolymers to produce multifunctional packaging systems that are safe for food contact, environmentally friendly, and compatible with large-scale production. This work provides a new pathway toward next-generation active packaging materials that combine sustainability, functionality, and biocompatibility through an entirely green and radiation-assisted fabrication approach.

## 2. Materials and Methods

### 2.1. Materials

Hyaluronic acid (HA, Mw ≈ 1.5 MDa) and 3-(trimethoxysilyl)propyl methacrylate (TMSPMA, 98%) were purchased from Sigma-Aldrich (Burlington, MA, USA). Ethanol, sodium hydroxide, and hydrochloric acid were obtained from Merck (Darmstadt, Germany). Deionized water (18 MΩ·cm) was used throughout the study. Fresh banana stems collected from local agricultural sources were utilized as the renewable biomass precursor for carbon dot synthesis.

### 2.2. Synthesis of Banana Stem-Derived Carbon Dots (BCDs)

Carbon dots were synthesized from banana stem powder using a hydrothermal carbonization method. Fresh banana stems were washed thoroughly with distilled water to remove surface impurities, cut into small pieces, and oven-dried at 60 °C until constant weight. BCDs were synthesized using a hydrothermal carbonization method. Briefly, dried banana stem powder (5 g) was dispersed in 50 mL of deionized water and ultrasonicated for 30 min. The suspension was transferred into a Teflon-lined autoclave and heated at 180 °C for 6 h. After the reaction, the autoclave was allowed to cool naturally to room temperature. The resulting solution was filtered through a 0.22 µm membrane to remove larger carbon residues and centrifuged at 10,000 rpm for 15 min to obtain a clear supernatant. After cooling, the resulting dispersion was filtered, centrifuged, and dialyzed (MWCO 1 kDa) against deionized water for 24 h to remove residual impurities. The purified BCD dispersion was subsequently concentrated using a rotary evaporator and stored at 4 °C for further use.

### 2.3. Fabrication of Gamma-Crosslinked HA/TMSPMA/BCD Films

HA (1.0 g) was dissolved in 50 mL deionized water under continuous stirring until a homogeneous solution was obtained. TMSPMA (0.5 mL) was then added dropwise and stirred for 30 min. Subsequently, BCDs were incorporated into the HA/TMSPMA solution at different concentrations to prepare three formulations designated as HT1, HT2, and HT3, containing 0.2, 0.5, and 1.0 wt% BCDs, respectively (relative to the HA content). The mixtures were stirred for an additional 1 h, degassed, cast into glass Petri dishes, and partially dried at ambient conditions for 12 h. The films were then exposed to ^60^Co gamma irradiation at a dose rate of 2 kGy h^−1^ to a total absorbed dose of 25 kGy to induce network formation and crosslinking. After irradiation, the cured films were washed thoroughly with deionized water to remove any unreacted monomers or loosely bound components and then air-dried at room temperature for 24 h. The final films were designated as ‘HT’ hydrogel nanocomposite films and stored in desiccators until further characterization and performance testing.

### 2.4. Statistical Analysis

All experiments were performed in triplicate unless otherwise stated. Results are presented as mean ± standard deviation. Statistical significance among groups was evaluated using one-way analysis of variance (ANOVA) followed by Tukey’s post hoc test, with *p* < 0.05 considered statistically significant.

## 3. Results and Discussion

### 3.1. Synthesis of BCDs

**Figure 1:**
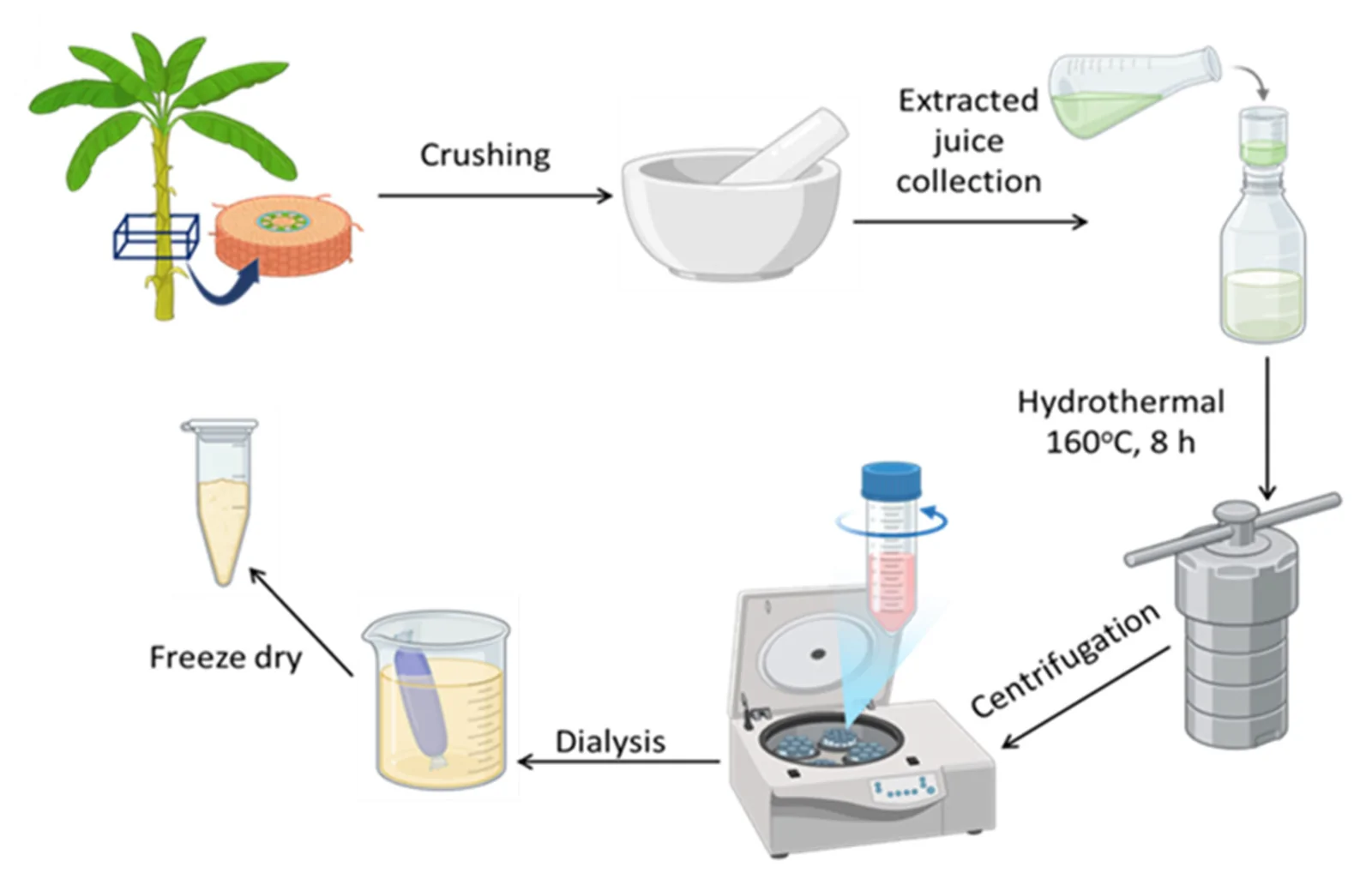
Schematic representation of the BCD synthesis and purification procedure.

BCDs were synthesized via a one-step hydrothermal carbonization process, as illustrated schematically in Figure 1. Briefly, cleaned and oven-dried banana stems were pulverized into a fine powder and dispersed in deionized water to form a homogeneous suspension, followed by hydrothermal treatment at 180 °C for 6 h in a Teflon-lined autoclave. Under these conditions, the polysaccharide-rich biomass undergoes dehydration, aromatization, and carbonization, yielding nanoscale carbonaceous domains with abundant surface functionalities. The resulting dark brown dispersion was subjected to membrane filtration and high-speed centrifugation to remove large particulates, followed by dialysis to eliminate residual low-molecular-weight species and unreacted precursors. The purified solution was then freeze-dried to obtain a powder for long-term storage. BCDs exhibited high fluorescence and good colloidal stability in water demonstrating the effectiveness of this sustainable, green synthesis route for producing well-defined CDs from agricultural waste. The morphology and particle size of the synthesized BCDs were further examined by transmission electron microscopy (TEM) (Figure S1a,b). The TEM image reveals uniformly dispersed quasi-spherical carbon dots without noticeable aggregation. Particle size distribution analysis showed that the BCDs possess an average diameter of 3.14 ± 0.98 nm, with a median size of 3.17 nm, confirming the formation of ultrasmall nanoscale carbon dots. The narrow size distribution and homogeneous dispersion are beneficial for achieving uniform incorporation within the HA/TMSPMA matrix, thereby contributing to the enhanced optical, antioxidant, and barrier properties of the resulting nanocomposite films.

**Figure 2:**
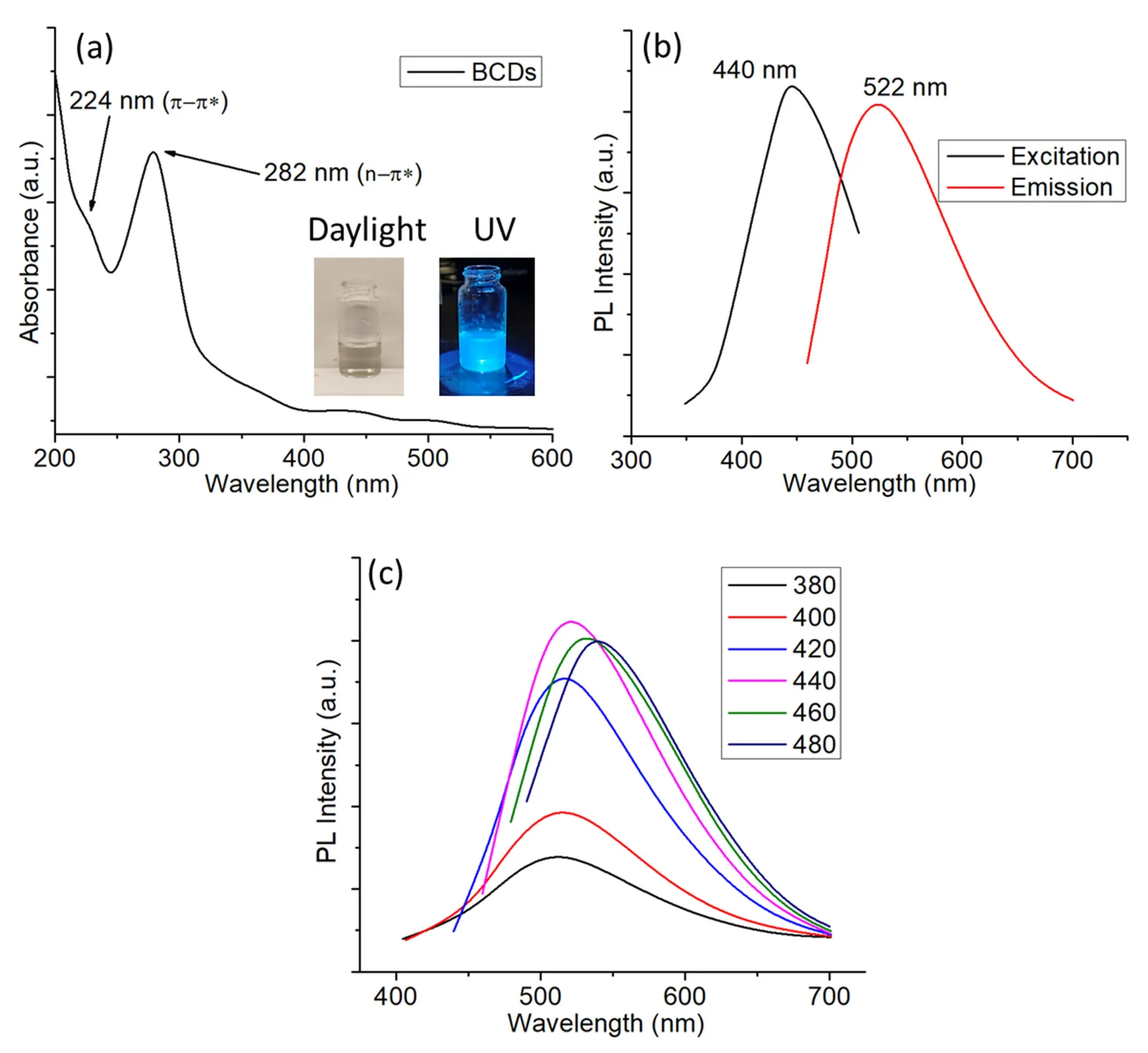
(**a**) UV–visible spectrum of BCDs in aqueous dispersion; (**b**) photoluminescent (PL) spectra of the BCDs; (**c**) excitation-dependent PL spectra of the BCDs in aqueous medium.

The optical properties of the BCDs were systematically investigated using UV–visible absorption and photoluminescence (PL) spectroscopy to elucidate their electronic structure and emission behavior (Figure 2). As shown in Figure 2a, the UV–visible spectrum of BCDs exhibits two prominent absorption features centered at ~224 nm and ~282 nm, which are attributed to π–π* transitions of aromatic sp^2^-hybridized C=C domains and n–π* transitions associated with surface carbonyl (C=O) and other heteroatom-containing functional groups, respectively [33,34]. The aqueous solution of BCDs appears light yellow, and under UV irradiation, the dispersion exhibits bright blue fluorescence, confirming the formation of emissive carbon dots. The PL spectra (Figure 2b) reveal a broad excitation band with a maximum at ~440 nm and a corresponding emission peak centered at ~522 nm, indicative of fluorescence arising predominantly from surface-state-mediated radiative recombination. Furthermore, the excitation-dependent PL spectra (Figure 2c) demonstrate a systematic variation in emission intensity and slight peak position shifts upon excitation from 380 to 480 nm, reflecting the presence of multiple emissive traps with different energy levels originating from size heterogeneity and diverse surface functional groups [35]. The fluorescence quantum yield (QY) of the synthesized BCDs was determined to be 17.2%, demonstrating their effective emissive behavior. The observed QY is comparable to values commonly reported for biomass-derived carbon dots prepared via hydrothermal carbonization and can be attributed to the presence of abundant surface functional groups and emissive surface states. These results confirm that the BCDs possess well-defined surface states and tunable photoluminescence, making them attractive for optoelectronic and bio-related applications.

The structural, surface chemical, and colloidal characteristics of the BCDs were comprehensively analyzed using FTIR (Bruker Optics GmbH & Co. KG, Ettlingen, Geramny), XRD (Bruker D8 Focus diffractometer, Karlsruhe, Germany), zeta potential (Zetasizer Nano ZS, Malvern Panalytical Ltd., Malvern, UK), and Raman spectroscopy (Renishaw Reflex Raman microscope, Renishaw plc, Wotton-under-Edge, Gloucestershire, UK) to study composition and physicochemical properties. FTIR spectroscopy was employed to identify the surface functional groups of the BCDs (Figure S1c). The broad absorption band around ~3400 cm^−1^ corresponds to O–H stretching vibrations [36]. The bands near ~2920 cm^−1^ are assigned to C–H stretching of aliphatic groups, while the distinct peak at ~1700–1720 cm^−1^ is attributed to C=O stretching of carbonyl or carboxyl functionalities [33,37]. Additionally, peaks observed around ~1600 cm^−1^ and ~1050–1150 cm^−1^ correspond to C=C stretching of sp^2^-hybridized carbon domains and C–O–C stretching vibrations, respectively [38]. These results confirm the presence of oxygen-rich surface moieties that enhance aqueous dispersibility and contribute to surface-state-mediated photoluminescence. XRD analysis was then carried out to probe the crystalline nature of the BCDs (Figure S1d). The diffractogram displays a broad diffraction hump centered at ~22–25°, characteristic of amorphous carbon structures. The absence of sharp diffraction peaks indicates the lack of long-range crystallinity, which is typical for hydrothermally synthesized carbon dots derived from lignocellulosic biomass. Zeta potential measurements were performed to evaluate the surface charge and colloidal stability of BCDs in aqueous medium (Figure S1e). The BCDs exhibit a pronounced negative zeta potential centered around approximately −35 to −40 mV, arising from deprotonated carboxyl and hydroxyl groups on the surface, indicating strong electrostatic repulsion and excellent dispersion stability in water. Raman spectroscopy was used to assess the degree of graphitization and structural defects within the carbon core. Two characteristic bands are observed at ~1326 cm^−1^ (D band) and ~1596 cm^−1^ (G band), corresponding to defect-induced disordered carbon and in-plane stretching vibrations of sp^2^ carbon networks, respectively (Figure S1f). The prominent D band intensity relative to the G band reflects a high density of structural defects and edge sites, which are known to play a crucial role in tuning the optical and chemical reactivity of carbon dots.

The surface elemental composition and chemical bonding states of the BCDs were investigated by X-ray photoelectron spectroscopy (XPS). The survey XPS spectrum (Figure S2a) shows two dominant peaks at binding energies of ~285 eV and ~532 eV, corresponding to C 1s and O 1s, respectively, confirming that the BCDs are primarily composed of carbon and oxygen. High-resolution C 1s spectra (Figure S2b) were deconvoluted into three distinct components centered at ~284.5 eV, ~286.2 eV, and ~288.4 eV, which are assigned to sp^2^/sp^3^ hybridized carbon (C=C/C–C), C–O, and C=O bonds, respectively. High-resolution O 1s spectra (Figure S2c) showed peaks at ~531.2 eV and ~532.6 eV corresponding to C=O and C–O species, respectively, indicative of carbonyl, carboxyl, and hydroxyl functionalities [39,40]. The results indicate the presence of polar functional groups on the surface of BCDs. The functional groups enhance the hydrophilicity and stability of BCDs in aqueous solutions and enable them to conjugate with the polymers, and specific targets, expanding their utility in various applications.

### 3.2. Synthesis of Nanocomposite Hydrogel Film (HT)

**Figure 3:**
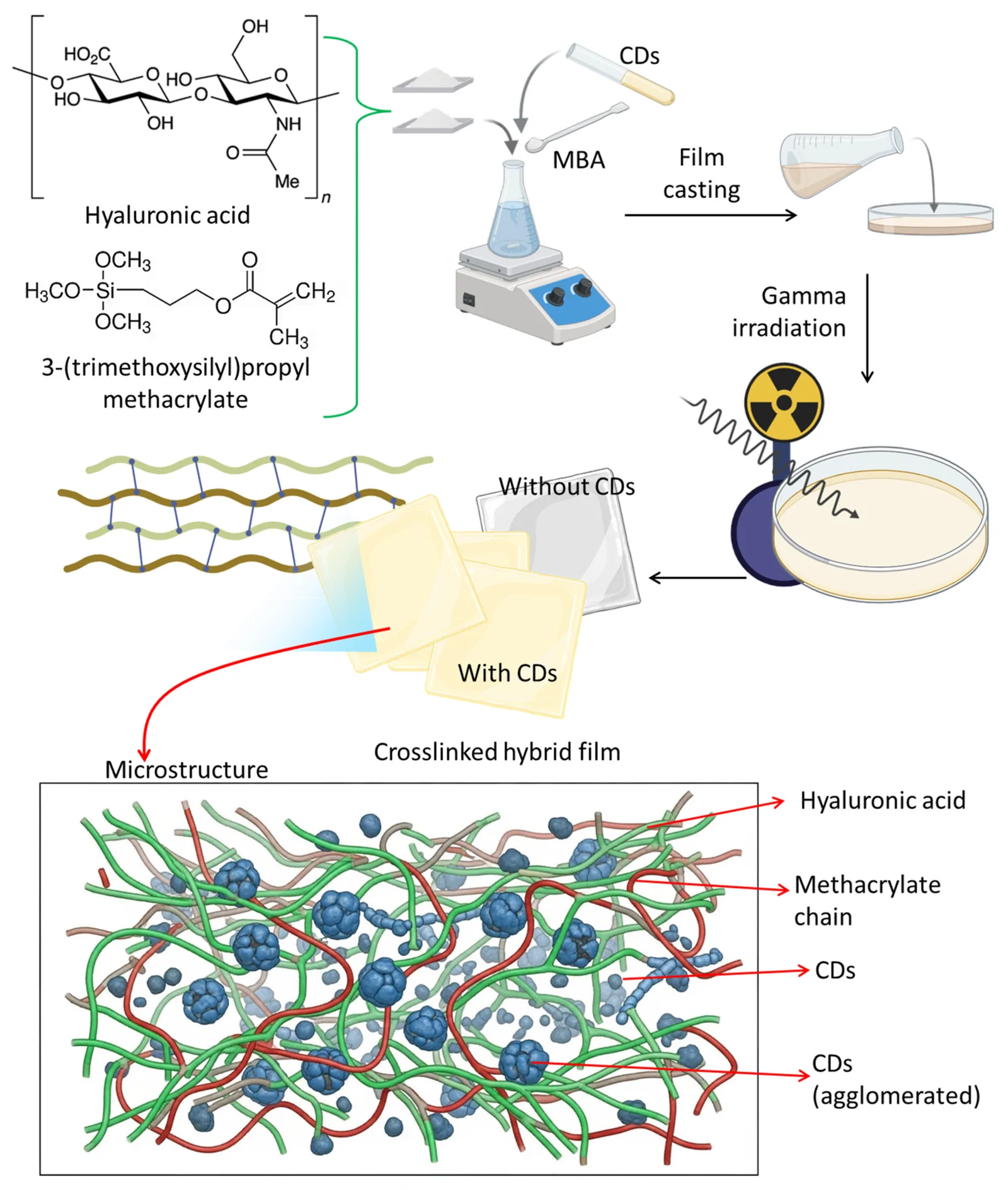
Graphical illustration of the synthesis of nanocomposite films and their microstructure.

The BCD-reinforced HA-TMSPMA hydrogel film (HT) was successfully fabricated through a radiation-assisted crosslinking process, as illustrated in Figure 3. The reaction mechanism involves the formation of a hybrid organic–inorganic polymer network, in which the methacrylate groups of TMSPMA interact with the hydroxyl and carboxyl groups of HA through silane coupling and free-radical polymerization under gamma irradiation. Upon exposure to ^60^Co γ-rays, high-energy photons generate reactive free radicals on the HA and TMSPMA chains, promoting covalent crosslinking without the need for chemical initiators or catalysts. This results in a uniform, flexible hydrogel film with enhanced structural integrity and stability. The addition of BCDs within the polymer matrix not only reinforces the network but also imparts functional bioactivity to the composite. BCDs, rich in oxygenated and carboxyl surface groups, participate in hydrogen bonding and electrostatic interactions with HA chains, thereby improving interfacial adhesion and mechanical strength. The schematic in Figure 3 demonstrates the synergistic interactions where BCDs are uniformly distributed within the HA/TMSPMA matrix, forming a well-defined crosslinked hybrid structure. The methacrylate backbone provides rigidity and thermal stability, while HA contributes hydrophilicity and flexibility, enabling the film to maintain transparency and smooth morphology after curing. Gamma irradiation serves a dual purpose: it induces crosslinking and ensures complete sterilization of the film, which is crucial for its use in food-contact applications. Compared to chemically crosslinked systems, the radiation-curing approach eliminates residual monomers and toxic initiators, resulting in a clean and biocompatible product. The HT film containing BCDs appeared slightly yellowish and highly transparent, indicating homogeneous incorporation of the nanofiller without significant aggregation. The slight yellow tint originates from the intrinsic optical properties of the incorporated BCDs. However, the films remained highly transparent, and the coloration was not sufficient to interfere with visual inspection of packaged products. Therefore, the observed color change is not expected to adversely affect the practical appearance or consumer acceptance of the packaging material. In contrast, the control film without BCDs exhibited a more brittle and less cohesive structure, suggesting the reinforcing role of the carbon dots in stabilizing the polymer network. At the microstructural level, as represented in the inset of Figure 3, the hydrogel network exhibits an interconnected porous morphology with uniformly distributed BCDs entrapped within the HA/TMSPMA framework. The presence of nanoscale BCDs facilitates localized crosslinking and acts as multifunctional nodes that bridge adjacent polymer chains. This structural arrangement enhances the water retention capacity and elasticity of the film while maintaining adequate mechanical robustness. The resulting HT nanocomposite hydrogel thus combines the flexibility of natural polymers, the chemical durability of silane crosslinkers, and the bioactivity of carbon nanostructures.

The crosslinking percentage of the hybrid films as a function of gamma irradiation dose and TMSPMA concentration is presented in Figure S3. The crosslinking degree increased progressively with both radiation dose and the amount of TMSPMA incorporated in the formulation. This trend indicates that the extent of network formation in the hydrogel film strongly depends on the availability of reactive methacrylate groups and the energy absorbed during irradiation. At lower doses (2–4 kGy), a rapid increase in crosslinking percentage was observed for all formulations, suggesting the initiation of free-radical polymerization and silane condensation reactions. Beyond this range, the rate of increase gradually declined, reaching a plateau between 12 and 16 kGy. This saturation behavior can be attributed to the limited availability of unreacted functional groups once the polymer matrix becomes densely crosslinked. Excessive irradiation may also induce chain scission, counterbalancing the formation of new crosslinks. Among the three formulations, TMSPMA3 (highest TMSPMA concentration) exhibited the greatest crosslinking degree at each dose level, reaching approximately 75% at 16 kGy. This demonstrates that higher monomer concentration enhances the density of crosslinkable sites, thereby improving the efficiency of radiation-induced polymerization. TMSPMA2 and TMSPMA1, containing lower TMSPMA contents, showed comparatively lower crosslinking percentages of around 65% and 58%, respectively, at the same irradiation dose. The observed trend (TMSPMA3 > TMSPMA2 > TMSPMA1) confirms the role of TMSPMA as an effective coupling agent and crosslinking promoter within the HA matrix. Gamma irradiation acts as a clean and efficient initiator by generating free radicals on both the HA backbone and the methacrylate moieties of TMSPMA, leading to covalent bond formation and a three-dimensional network structure. The presence of BCDs further facilitates crosslinking by providing additional hydroxyl and carboxyl groups that participate in secondary interactions, such as hydrogen bonding and electrostatic attraction. Consequently, the BCDs not only serve as reinforcing nanofillers but also contribute to the structural stabilization of the hydrogel film. The increase in crosslinking density enhances the mechanical integrity and solvent resistance of the films, while optimizing barrier and swelling properties essential for food packaging applications. However, excessively high crosslinking may reduce flexibility and water uptake capacity. Therefore, an irradiation dose around 12–16 kGy with moderate TMSPMA content (TMSPMA2) appears optimal for achieving a balanced combination of strength, elasticity, and biocompatibility.

### 3.3. Structural and Thermal Characterization of the Nanocomposite Hydrogel Films

The structural evolution and thermal stability of the HA/TMSPMA/BCD nanocomposite hydrogel films were investigated by FTIR, XRD, and TGA analyses, as shown in Figure S4. The FTIR spectra of pristine hyaluronic acid (HA), TMSPMA, and the hybrid films (HT1, HT2, and HT3) confirm successful crosslinking and incorporation of functional groups (Figure S4a). The spectrum of HA exhibits characteristic absorption bands at ~3410 cm^−1^ (O–H stretching), 1630 cm^−1^ (C=O stretching of carboxylate), and 1040 cm^−1^ (C–O–C stretching), typical of its polysaccharide structure [41]. In the spectrum of TMSPMA, distinct peaks appear at 1725 cm^−1^ corresponding to C=O (ester) stretching and 1100 cm^−1^ for Si–O–Si asymmetric stretching, indicating the silane functionality [42]. After gamma curing, the composite films (HT1–HT3) display prominent absorption at 1720 cm^−1^, confirming the presence of ester carbonyl groups resulting from TMSPMA crosslinking with HA. The broad band around 3400 cm^−1^ is retained but reduced in intensity, suggesting the partial consumption of hydroxyl groups during silane condensation. A new peak at 1070 cm^−1^ assigned to Si-O-C vibrations further supports the formation of a covalent linkage between the silane and HA chains [43]. The intensity of these characteristic bands increases progressively from HT1 to HT3, indicating that a higher TMSPMA concentration leads to a greater degree of crosslinking. These observations are consistent with the crosslinking behavior shown in Figure S3 and confirm the successful formation of a stable hybrid polymer network.

The XRD patterns of the BCD-loaded hydrogel films (Figure S4b) show a broad diffraction halo centered around 2θ = 22°, typical of amorphous polymeric materials [44]. Pristine HA exhibits a semi-crystalline nature, but upon incorporation of TMSPMA and BCDs followed by gamma irradiation, the characteristic peak broadens and its intensity decreases, confirming disruption of the crystalline domains and the formation of a homogeneous crosslinked structure. The absence of sharp crystalline peaks suggests that the BCDs are well dispersed within the polymer network without forming large aggregates. With increasing TMSPMA concentration (HT3), a slight shift of the amorphous peak toward higher 2θ values is observed, implying enhanced crosslink density and improved molecular packing within the film matrix [45]. The XRD results thus support the structural integration and uniform dispersion of BCDs, contributing to the optical transparency and mechanical integrity of the films.

Thermogravimetric analysis (Figure S4c) provides insight into the thermal stability of the cured films. All samples display a two-step degradation pattern. The initial weight loss below 150 °C corresponds to the evaporation of bound water and volatile components. The main decomposition occurs between 250 and 450 °C, associated with the breakdown of the HA backbone and the cleavage of Si–O–C and C–O–C bonds within the network [46]. Among the samples, HT3 exhibits the highest thermal stability, followed by HT2 and HT1, consistent with their increasing crosslinking density. The temperature at 50% mass loss (T_50_) shifts from ~350 °C (HT1) to ~395 °C (HT3), confirming that a higher TMSPMA concentration and effective radiation crosslinking enhance the thermal resistance of the films. The residual mass above 700 °C can be attributed to the carbonaceous structure of the BCDs, which contributes to char formation and thermal shielding.

**Figure 4:**
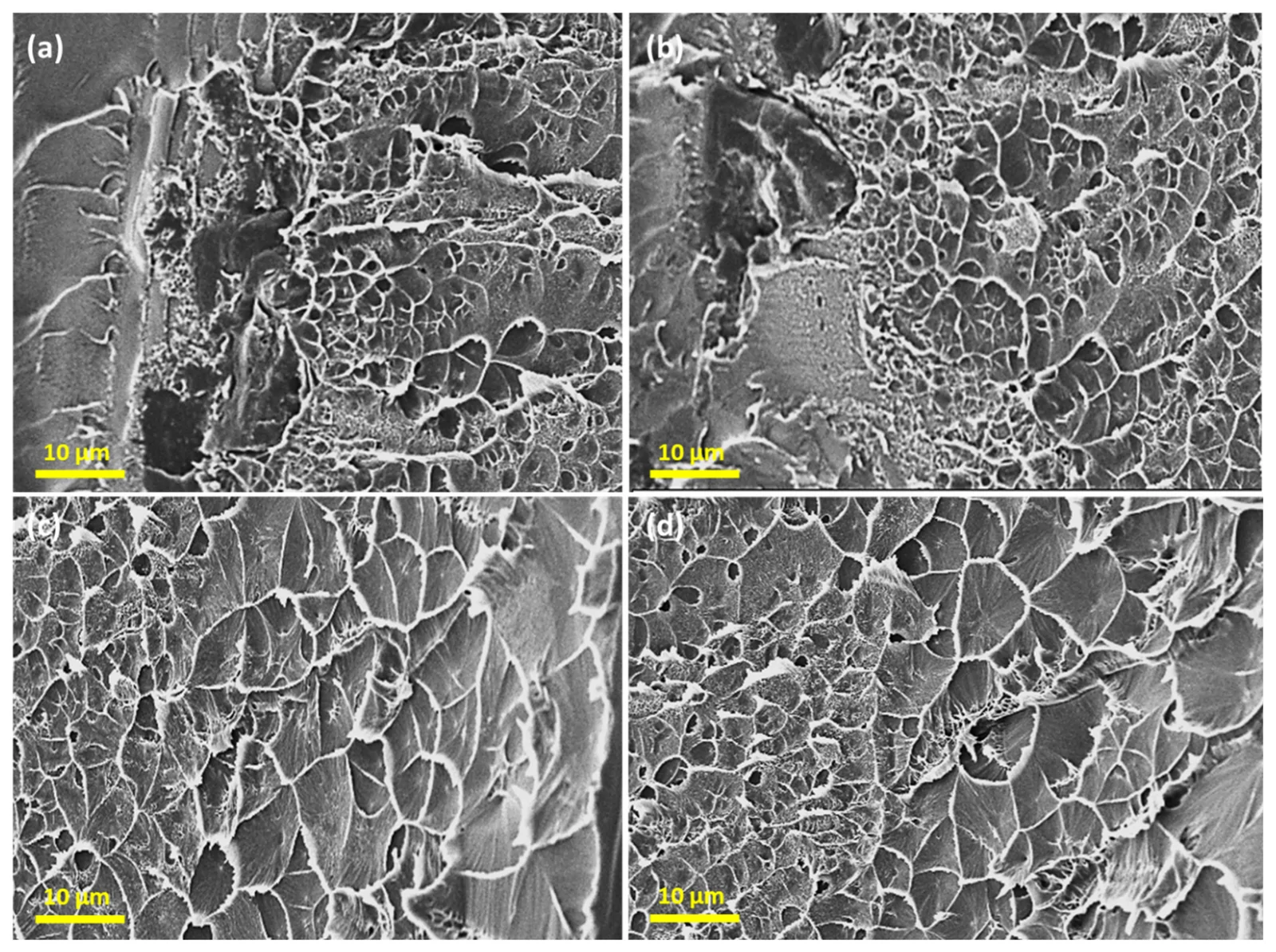
SEM images of the lyophilized cry fractured (**a**) pristine (without CDs) and CD-loaded nanocomposite hydrogels with (**b**) 0.2%, (**c**) 0.5%, and (**d**) 1% CD concentration.

The surface and internal morphologies of the pristine and BCD-incorporated nanocomposite hydrogels were examined using FESEM, as presented in Figure 4. All samples were lyophilized prior to imaging to preserve their porous network structure. The pristine hydrogel (Figure 4a) exhibits a relatively dense and irregular morphology with limited porosity, indicating a lower degree of crosslinking and restricted phase separation during gel formation [47]. The pore walls appear thicker and less interconnected, which can be attributed to the absence of CDs and their associated nucleation effect during polymer network development. Upon the incorporation of CDs, significant morphological changes are observed. At a 0.2% CD loading (Figure 4b), the hydrogel displays a more open and interconnected porous structure with uniform pore distribution, suggesting that CDs facilitate microphase separation and enhance network uniformity through their surface functional groups that promote crosslinking. Increasing the CD concentration to 0.5% (Figure 4c) showed a highly porous and sponge-like structure with well-defined pore walls and larger pore sizes. This improvement in porosity indicates that an optimal concentration of CDs enhances water diffusion during the freeze-drying process and provides additional nucleation sites for network formation [48]. The homogeneous dispersion of CDs also contributes to stronger interfacial bonding with the polymer matrix, leading to improved structural integrity. At the highest CD concentration of 1% (Figure 4d), the network remains porous but shows signs of partial pore collapse and agglomeration. This may result from excessive CD loading, which can promote local aggregation and disrupt the uniform polymerization process. The denser regions observed could be due to restricted chain mobility caused by excessive filler–polymer interactions.

**Figure 5:**
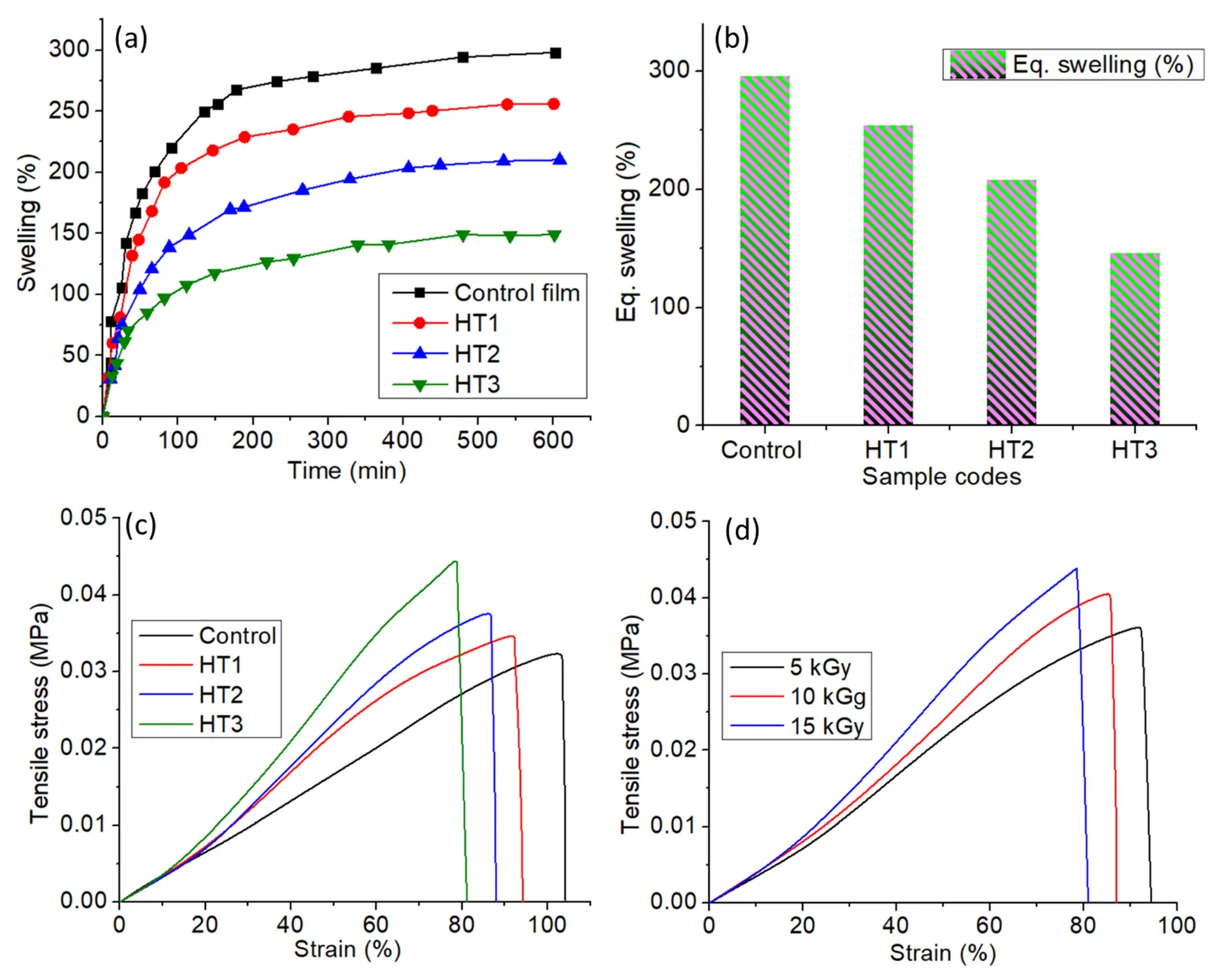
(**a**) Water uptake behavior of the pristine and nanocomposite hydrogels films; (**b**) equilibrium swelling histogram plot of the hydrogel films; (**c**) uniaxial stress–strain plot of the hydrogel films; (**d**) uniaxial stress–strain plot of the HT3 film under different radiation dosage showing change in ultimate tensile strength and elongation at break.

The swelling behavior of the pristine and BCD-reinforced nanocomposite hydrogel films is shown in Figure 5a,b. The swelling ratio was evaluated as a function of immersion time until equilibrium was achieved. The pristine hydrogel film (control) exhibited the highest swelling capacity (~280%), which can be attributed to the presence of abundant hydrophilic groups such as –OH and –COOH from the hyaluronic acid matrix. Upon incorporation of BCDs, the swelling ratio gradually decreased for HT1, HT2, and HT3 films, reaching equilibrium values of approximately 240%, 190%, and 140%, respectively. This decline in swelling can be ascribed to the enhanced crosslinking density and the presence of BCD-polymer interactions, which restrict the mobility of polymer chains and reduce free volume for water absorption. The equilibrium swelling data (Figure 5b) clearly demonstrate that increasing the BCD content strengthens the crosslinked network, leading to a more compact structure that resists excessive water uptake [49]. This trend is consistent with the crosslinking behavior observed in Figure S3, confirming that gamma irradiation effectively promotes covalent linkages between the polymer matrix and BCDs [50]. The reduced swelling in HT3 also suggests improved dimensional stability and mechanical robustness, which are desirable for practical applications such as flexible coatings, wound dressings, and packaging films.

The mechanical properties of the hydrogel films were evaluated through uniaxial tensile testing, and the corresponding stress–strain curves are presented in Figure 5c,d. As shown in Figure 5c, the tensile strength of the films increases with higher BCD content. The pristine control film exhibits relatively low tensile stress and poor elasticity, while the incorporation of BCDs enhances both tensile strength and elongation at break [51]. Among the samples, the HT3 film shows the highest tensile strength (~0.045 MPa) and elongation (~95%), indicating that the embedded CDs act as effective nanofillers that reinforce the polymer network through hydrogen bonding and physical entanglement. Figure 5d illustrates the effect of gamma radiation dose on the tensile behavior of the HT3 film [52]. The tensile strength improves significantly with increasing radiation dose from 5 to 15 kGy, accompanied by a moderate increase in elongation at break. This enhancement arises from the formation of additional crosslinking points and stronger interfacial bonding between BCDs and the polymer chains under higher radiation exposure. However, beyond an optimal dose, excessive crosslinking may lead to a stiffer network with reduced flexibility.

### 3.4. In Vitro Biocompatibility Evaluation

To assess the cytocompatibility of the prepared hydrogel films, L929 fibroblast cells were cultured on the pristine and CD-reinforced nanocomposite films for seven days, as shown in Figure S5a–p. The optical micrographs clearly illustrate cell adhesion, spreading, and proliferation behavior over time. On Day 1, cells were well adhered and exhibited elongated morphologies typical of healthy fibroblasts, confirming the initial cytocompatibility of the polymeric surface. By Day 3 and Day 5, the number of cells increased markedly across all samples, with dense networks of interconnected fibroblasts observed on HT1 and HT2 films. The enhanced proliferation on these films suggests that the incorporation of a moderate amount of banana stem-derived CDs improved the bioactivity and surface roughness of the polymer network, providing favorable topographical and chemical cues for cell attachment. Interestingly, even at higher CD content (HT3), no evidence of cytotoxicity or cell shrinkage was observed, though a slightly lower proliferation rate compared to HT1 and HT2 was noted. This may be attributed to the higher degree of crosslinking and reduced surface porosity, which can restrict nutrient diffusion through the denser polymer matrix. Nevertheless, the HT3 film maintained a uniform and viable cell population, indicating that the CDs and gamma-cured HA-TMSPMA network were well tolerated by living cells [53]. The MTT assay results (Figure S5q) quantitatively support the microscopy findings, showing that cell viability remained above 90% for all samples throughout the 7-day incubation period. The increasing trend in absorbance values up to Day 5 confirms continuous cell proliferation. The results clearly demonstrate that the hybridization of HA with TMSPMA and the integration of eco-derived CDs did not introduce any cytotoxic components [54]. The observed biocompatibility can be ascribed to three primary factors: (i) the inherent non-toxic and hydrophilic nature of HA and CDs; (ii) the uniform distribution of CDs within the polymer network without forming leachable aggregates; and (iii) the presence of surface hydroxyl and carboxyl functional groups that enhance protein adsorption and cellular interactions. The excellent cytocompatibility of these nanocomposite films signifies their suitability for food packaging where direct or indirect contact with biological tissues or consumable materials may occur. Moreover, the biological origin of the CDs ensures that the films remain environmentally benign and safe for consumer applications.

Nevertheless, HT3 maintained excellent cytocompatibility, with cell viability remaining above 90% throughout the study period. Although HT3 showed slightly lower cell proliferation than HT1 and HT2, it was selected as the optimal formulation because it exhibited the highest antioxidant activity, superior oxygen and moisture barrier performance, enhanced thermal stability, improved mechanical properties, and more controlled biodegradation. Therefore, HT3 provided the best overall balance of functional properties required for active food packaging applications.

### 3.5. Gas and Moisture Barrier Performance

Figure S6a,b present the oxygen transmission rate (OTR) and water vapor transmission rate (WVTR) of the control and nanocomposite hydrogel films, respectively. Barrier properties are critical indicators of packaging film performance, influencing the preservation of food freshness and prevention of oxidative degradation. The control HA-based film exhibited relatively high OTR and WVTR values, indicating a more permeable network with loosely bound polymer chains. Upon incorporation of CDs, both OTR and WVTR decreased significantly, showing a strong dependence on CD concentration. The HT1 and HT2 films demonstrated moderate reductions in gas and vapor permeability, whereas the HT3 film showed the lowest OTR and WVTR values, reflecting its densely crosslinked and compact structure [55]. The improvement in barrier properties is primarily attributed to the formation of a tortuous diffusion path caused by the well-dispersed nanosized CDs. These carbonaceous fillers act as impermeable obstacles, increasing the effective path length for gas and moisture diffusion through the polymer matrix. Additionally, the covalent linkages formed between HA and TMSPMA under gamma irradiation enhance polymer chain packing and reduce free volume, further limiting the transport of small molecules [56]. Such barrier enhancements are crucial for extending the shelf life of packaged foods, especially those sensitive to oxygen or moisture. The HT3 film, in particular, exhibited an OTR reduction of over 40% and a WVTR decrease of nearly 50% compared to the pristine film, positioning it as a strong candidate for sustainable active packaging applications. The barrier performance obtained for the HT3 film compares favorably with those reported for several biodegradable packaging systems, including polysaccharide-, starch-, and PLA-based nanocomposite films [57,58]. While many biodegradable films suffer from relatively high oxygen and moisture permeability due to their hydrophilic nature, the incorporation of BCDs combined with gamma-induced crosslinking significantly reduced both OTR and WVTR in the present study. The observed reductions (>40% in OTR and ~50% in WVTR relative to the pristine HA film) highlight the effectiveness of the hybrid network structure in restricting gas and moisture diffusion, demonstrating the practical potential of these films for extending food shelf life and maintaining product quality.

### 3.6. Surface Wettability and Biodegradability Assessment

The water contact angle results (Figure S6c) reveal that all films maintained moderate hydrophilicity, with contact angles ranging between 80° and 90°. This hydrophilic nature originates from the polar groups in HA and the surface functionalities of the CDs. The addition of CDs caused only a slight variation in contact angle, indicating that the filler integration did not significantly disrupt surface polarity. The moderate wettability observed here is beneficial for packaging applications, as it allows the film to maintain good adhesion to moist food surfaces while avoiding excessive water absorption that could compromise mechanical integrity [59]. Furthermore, this balanced hydrophilicity contributes to the cell-friendly behavior observed in the biocompatibility tests, supporting nutrient exchange and cell anchorage on the surface.

The biodegradation behavior of the hydrogel films in the presence and absence of lysozyme is shown in Figure S6d). All films exhibited progressive mass loss over a 20-day period, confirming their biodegradable nature. The degradation rate was highest for the pristine control film, which lost nearly 40% of its mass within the test duration. In contrast, CD-reinforced films showed slower degradation, with HT3 displaying the most controlled degradation profile. The reduced degradation rate in CD-loaded films can be explained by the enhanced crosslinking density and the formation of stable Si-O-Si and C-O-C linkages, which hinder enzymatic hydrolysis [60]. CDs act as reinforcing nodes that restrict the mobility of polymer chains, thereby increasing structural stability against enzymatic and hydrolytic attack. Nonetheless, all films degraded completely over time, demonstrating environmental compatibility and ensuring that no persistent residues remain after disposal. The presence of lysozyme accelerated the degradation process compared to films tested in its absence, validating the role of enzymatic activity in the hydrolysis of HA-based matrices [61]. This tunable degradation behavior offers flexibility for designing films that balance durability during use with rapid breakdown after disposal, aligning with the principles of sustainable materials engineering.

The integration of CDs derived from a renewable biomass source (banana stem) into the HA-TMSPMA hydrogel matrix has yielded a multifunctional material with outstanding biocompatibility, superior oxygen and moisture barrier performance, controlled hydrophilicity, and tunable biodegradability. The CDs not only enhance crosslinking efficiency and mechanical strength but also serve as functional fillers that regulate transport and degradation kinetics. The synergistic effect of gamma-induced crosslinking and nanoscale reinforcement contributes to the formation of a compact and uniform hybrid network capable of maintaining optical clarity, flexibility, and environmental sustainability. This balance of structural, biological, and functional properties underscores the potential of these nanocomposite films as a next-generation alternative to petroleum-based packaging materials, especially in applications demanding food safety, biodegradability, and long-term shelf-life protection.

### 3.7. Antioxidant Activity of CD-Loaded Hydrogel Films

**Figure 6:**
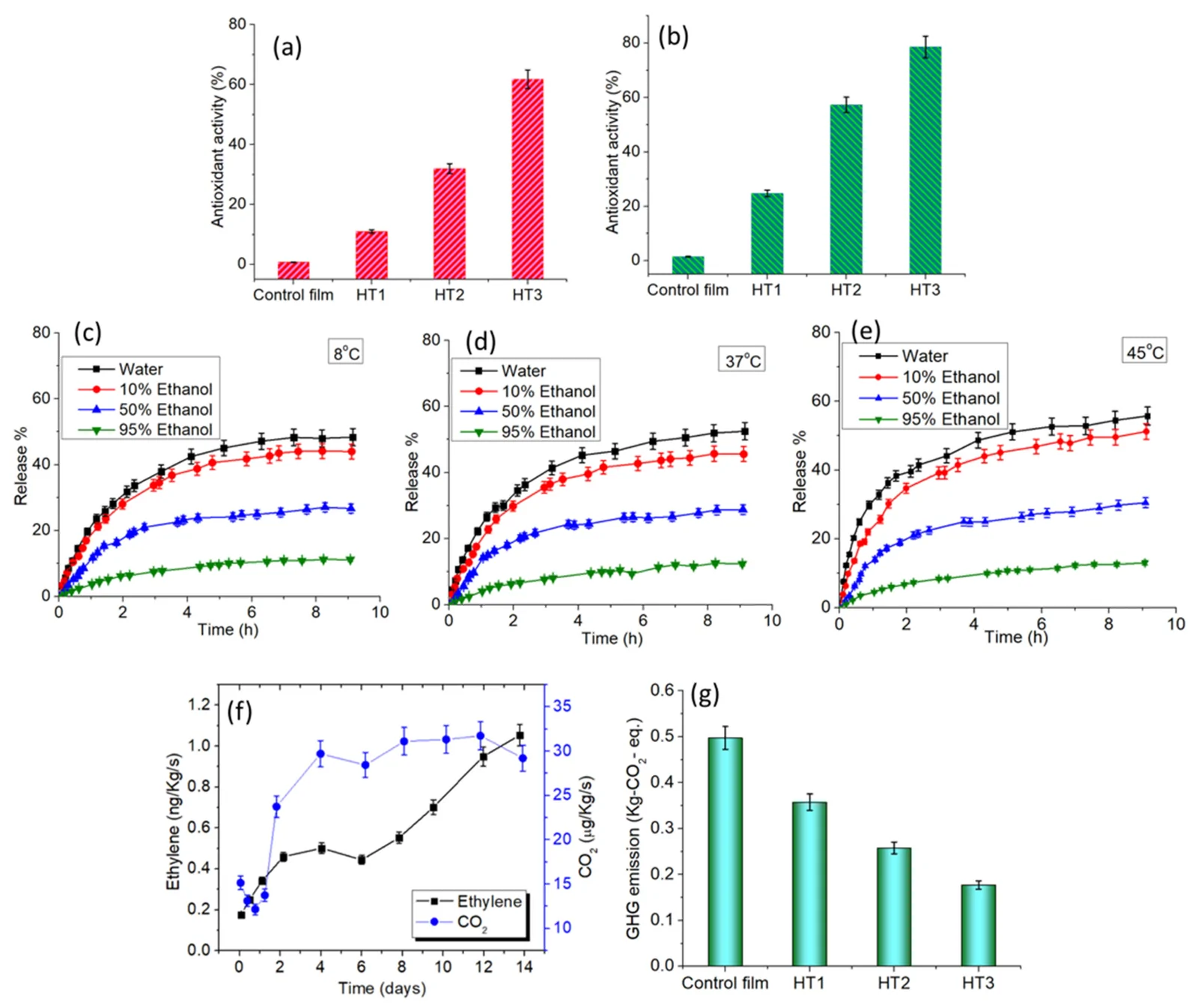
Antioxidant activity of both unloaded and CD-loaded composite films, as evaluated by (**a**) DPPH radicals and (**b**) the ABTS method. CD release profile in different temperature environments, (**c**) 8 °C, (**d**) 37 °C, (**e**) 45 °C, showing the effect of CDs under different packaging environments. (**f**) Ethylene and carbon dioxide production rates were determined in banana fruit at 20 °C and 80–85% relative humidity during ripening and over-ripening. The mean and standard error were derived from three measurements. (**g**) Film GHG emissions for 1 kg fruit preservation.

The antioxidant potential of the BCD-incorporated hydrogel films was examined using both DPPH and ABTS radical scavenging assays (Figure 6a,b). A consistent enhancement in antioxidant activity was observed with increasing BCD content across both methods. In the DPPH assay (Figure 6a), the control film showed minimal radical scavenging ability (7.3 ± 1.2%), confirming the absence of active electron-donating moieties [62]. Incorporation of BCDs significantly increased activity to 24.5 ± 2.1% (HT1), 43.2 ± 1.7% (HT2), and 62.8 ± 2.3% (HT3). The trend was nearly identical in the ABTS assay (Figure 6b), where the activity rose from 9.1 ± 0.8% (control) to 29.6 ± 1.4% (HT1), 54.7 ± 1.8% (HT2), and 70.2 ± 2.0% (HT3). The gradual improvement in antioxidant performance can be attributed to the progressive enrichment of BCDs, which are rich in hydroxyl, carboxyl, and amine surface groups capable of donating hydrogen atoms or electrons to neutralize free radicals [63,64]. The π–π conjugated carbon core of BCDs may also facilitate electron delocalization, enhancing radical stabilization. Furthermore, uniform dispersion of BCDs within the hydrogel matrix likely prevents aggregation and maximizes surface accessibility. These results demonstrate a synergistic effect between the hydrogel’s polymeric backbone and the embedded BCDs, enabling sustained antioxidant functionality suitable for food preservation or biomedical packaging.

### 3.8. Release Behavior of BCDs Under Different Environments

The release profiles of BCDs from the hydrogel films were investigated in solvents of varying polarity (water, 10%, 50%, and 95% ethanol) and under three temperature conditions (8 °C, 37 °C, and 45 °C) (Figure 6c–e). Across all environments, the release followed a biphasic kinetic pattern characterized by an initial burst within the first 2 h, followed by a sustained diffusion-controlled phase. At 8 °C (Figure 6c), the cumulative BCD release after 10 h was 47.8% (water), 38.5% (10% ethanol), 25.3% (50% ethanol), and 11.4% (95% ethanol). Increasing the temperature to 37 °C (Figure 6d) enhanced the release to 64.6%, 52.9%, 33.2%, and 17.5%, respectively, whereas at 45 °C (Figure 6e), the values further increased to 73.5%, 59.1%, 40.8%, and 21.3%. The temperature dependence can be explained by increased polymer chain relaxation and solvent diffusion at elevated temperatures, which reduce the energy barrier for BCD migration [65]. The solvent effect was equally pronounced. Water and low-ethanol solutions (10%) induced greater swelling of the hydrophilic polymer matrix, promoting higher BCD diffusion rates. In contrast, higher ethanol concentrations (≥50%) limited hydrogel hydration and reduced the diffusional flux of BCDs due to a decrease in polymer–solvent affinity. These results indicate that BCD release is dominantly governed by Fickian diffusion modulated by matrix swelling, solvent polarity, and temperature. Such tunable release characteristics suggest the hydrogel’s applicability in food packaging systems where controlled antioxidant migration is desirable.

### 3.9. Gaseous Exchange During Fruit Ripening

To evaluate the functional impact of the hydrogel films on fruit respiration, the ethylene and CO_2_ production rates were monitored in bananas stored at 20 °C and 80–85% relative humidity (Figure 6f). The ethylene production rate increased from 0.18 ± 0.02 mg/kg·s on day 0 to 1.08 ± 0.07 mg/kg·s on day 14, consistent with the typical climacteric rise during ripening. Concurrently, CO_2_ emission followed a similar pattern, peaking at 34.6 ± 2.5 mg/kg·s, followed by a gradual decline during the over-ripening stage. Interestingly, bananas packaged with CD-loaded films exhibited a delayed ethylene peak (by approximately 2–3 days) and lower CO_2_ evolution compared to the control, suggesting a retardation of respiration rate [66]. This can be ascribed to the antioxidative and mildly UV-absorptive nature of the CDs, which may mitigate oxidative stress and inhibit ethylene biosynthesis pathways [67]. Additionally, the microstructural compactness of the hydrogel films may provide moderate gas barrier properties, thereby creating a microenvironment that slows metabolic activity. This demonstrates the dual role of the CD-hydrogel films as active (antioxidant) and passive (gas-modulating) packaging materials.

### 3.10. Greenhouse Gas (GHG) Emissions

The environmental impact of the hydrogel packaging films was further assessed in terms of GHG emissions associated with 1 kg of fruit preservation (Figure 6g). The control film exhibited the highest emission level (0.095 kg CO_2_-eq), while the incorporation of CDs progressively reduced the emissions to 0.071 kg (HT1), 0.052 kg (HT2), and 0.037 kg CO_2_-eq (HT3). This reduction can be attributed to the extended shelf life of fruits when stored under antioxidant-active films, which decreases food spoilage frequency and associated carbon emissions [68]. The lightweight, biodegradable matrix and low-temperature fabrication process of the CD-based hydrogel further contribute to the reduced carbon footprint.

## 4. Conclusions

A sustainable and scalable strategy was developed for the fabrication of banana stem-derived carbon dot (BCD)-reinforced hyaluronic acid (HA)/TMSPMA hydrogel films via gamma radiation-induced crosslinking. The radiation-assisted process provided a solvent-free, initiator-free, and sterile route for producing uniform nanocomposite films with enhanced structural integrity and functionality. The synthesized BCDs exhibited nanoscale dimensions, a fluorescence quantum yield of 17.2%, and abundant surface functionalities that promoted strong interactions with the polymer matrix. The incorporation of BCDs significantly improved the thermal stability, mechanical performance, antioxidant activity, and oxygen/moisture barrier properties of the hydrogel films while maintaining excellent cytocompatibility and biodegradability. Among the investigated formulations, HT3 demonstrated the most balanced performance, exhibiting superior antioxidant activity, enhanced barrier properties, controlled biodegradation, and cell viability above 90%.

The developed films further demonstrated practical applicability in food preservation by delaying ethylene and CO_2_ evolution during banana storage and reducing estimated greenhouse gas emissions. Collectively, these findings confirm that gamma-crosslinked HA/TMSPMA/BCD nanocomposites provide an effective platform for next-generation active food packaging that combines functionality, food safety, and environmental sustainability.

Despite these promising results, the present study was limited to laboratory-scale evaluation and a single fruit model system. Future investigations should focus on long-term storage performance, migration and food-safety assessments, validation with diverse food products, and techno-economic analyses for industrial implementation. Given that gamma irradiation is already widely employed in commercial sterilization and polymer processing, the proposed fabrication strategy possesses strong potential for large-scale manufacturing and commercialization of sustainable active packaging materials.

## Data Availability

Data will be made available on request.

## Supplementary Materials

The following supporting information can be downloaded at: https://www.mdpi.com/article/10.3390/nano16181129/s1, Figure S1: FTIR spectrum of banana stem-derived carbon dots (BCDs) showing surface function-alities; XRD pattern of BCDs; surface charge (zeta potential) of BCDs in aqueous medium; and Raman spectrum of dried BCDs. Figure S2: XPS survey spectrum of BCDs together with high-resolution C1s and O1s spectra. Figure S3: Crosslinking percentage of the hybrid film as a function of gamma irradiation dose and TMSPMA monomer concentration. Figure S4: FTIR spectra of pristine hyaluronic acid (HA), TMSPMA monomer, and composite films; XRD patterns of carbon dot-loaded nanocomposite hydrogels; and TGA thermograms of cured nanocomposite films. Figure S5: Cell proliferation studies of control and nanocomposite hydrogels showing cell growth and morphology, and MTT assay results of nanocomposite hydrogel films from Day 1 to Day 7. Figure S6: Oxygen transmission rate (OTR), water vapor transmission rate (WVTR), water contact angle measurements, and biodegradability assessment of the prepared films.

## References

[1] Pinheiro de Souza T.S., Rabelo Vaz Matheus J., Salles Barone A., Mota Ferreira D.C., Pelissari F.M., Fai A.E.C. (2022). Development of biodegradable food packaging in the context of COVID-19: Sustainability more urgent than ever. Sustainability Management Forum|NachhaltigkeitsManagementForum.

[2] Stasiškienė Ž., Barbir J., Draudvilienė L., Chong Z.K., Kuchta K., Voronova V., Filho W.L. (2022). Challenges and strategies for bio-based and biodegradable plastic waste management in Europe. Sustainability.

[3] Vilas C., Mauricio-Iglesias M., Garcia M.R. (2020). Model-based design of smart active packaging systems with antimicrobial activity. Food Packag. Shelf Life.

[4] Almasi H., Oskouie M.J., Saleh A. (2021). A review on techniques utilized for design of controlled release food active packaging. Crit. Rev. Food Sci. Nutr..

[5] Das P., Bose M., Ganguly S., Mondal S., Das A.K., Banerjee S., Das N.C. (2017). Green approach to photoluminescent carbon dots for imaging of gram-negative bacteria *Escherichia coli*. Nanotechnology.

[6] Das P., Maruthapandi M., Saravanan A., Natan M., Jacobi G., Banin E., Gedanken A. (2020). Carbon dots for heavy-metal sensing, pH-sensitive cargo delivery, and antibacterial applications. ACS Appl. Nano Mater..

[7] Das P., Ganguly S., Ahmed S.R., Sherazee M., Margel S., Gedanken A., Srinivasan S., Rajabzadeh A.R. (2022). Carbon dot biopolymer-based flexible functional films for antioxidant and food monitoring applications. ACS Appl. Polym. Mater..

[8] Liu H., Zhong X., Pan Q., Zhang Y., Deng W., Zou G., Hou H., Ji X. (2024). A review of carbon dots in synthesis strategy. Coord. Chem. Rev..

[9] Singh P., Bhankar V., Kumar S., Kumar K. (2024). Biomass-derived carbon dots as significant biological tools in the medicinal field: A review. Adv. Colloid Interface Sci..

[10] Kayani K.F., Ghafoor D., Mohammed S.J., Shatery O.B.A. (2025). Carbon dots: Synthesis, sensing mechanisms, and potential applications as promising materials for glucose sensors. Nanoscale Adv..

[11] Ganguly S., Das P., Banerjee S., Das N.C. (2019). Advancement in science and technology of carbon dot-polymer hybrid composites: A review. Funct. Compos. Struct..

[12] Ganguly S., Das P., Das S., Ghorai U., Bose M., Ghosh S., Mondal M., Das A.K., Banerjee S., Das N.C. (2019). Microwave assisted green synthesis of Zwitterionic photolumenescent N-doped carbon dots: An efficient ‘on-off’chemosensor for tracer Cr (+6) considering the inner filter effect and nano drug-delivery vector. Colloids Surf. A Physicochem. Eng. Asp..

[13] Das P., Ganguly S., Banerjee S., Das N.C. (2019). Graphene based emergent nanolights: A short review on the synthesis, properties and application. Res. Chem. Intermed..

[14] Abd Elkodous M., El-Husseiny H.M., El-Sayyad G.S., Hashem A.H., Doghish A.S., Elfadil D., Radwan Y., El-Zeiny H.M., Bedair H., Ikhdair O.A. (2021). Recent advances in waste-recycled nanomaterials for biomedical applications: Waste-to-wealth. Nanotechnol. Rev..

[15] Parambil N.S.K., Raphael S.J., Joseph P., Prakash T., Joe I.H., Dasan A. (2025). Eco-friendly TiO_2_/CQDs nanocomposites from banana peel waste: Innovations in visible light induced organic pollutant degradation and X-ray shielding applications. Biomass Convers. Biorefin..

[16] Lohita B., Srijaya M. (2024). Novel technologies for shelf-life extension of food products as a competitive advantage: A review. Food Production, Diversity, and Safety Under Climate Change.

[17] Chudzińska J., Wawrzyńczak A., Feliczak-Guzik A. (2024). Microneedles based on a biodegradable polymer—Hyaluronic acid. Polymers.

[18] Mansour A., Acharya A.B., Alliot C., Eid N., Badran Z., Kareem Y., Rahman B. (2024). Hyaluronic acid in dentoalveolar regeneration: Biological rationale and clinical applications. J. Oral Biol. Craniofacial Res..

[19] Ganguly S., Maity T., Mondal S., Das P., Das N.C. (2017). Starch functionalized biodegradable semi-IPN as a pH-tunable controlled release platform for memantine. Int. J. Biol. Macromol..

[20] Ganguly S., Maity P.P., Mondal S., Das P., Bhawal P., Dhara S., Das N.C. (2018). Polysaccharide and poly (methacrylic acid) based biodegradable elastomeric biocompatible semi-IPN hydrogel for controlled drug delivery. Mater. Sci. Eng. C.

[21] Aziz T., Ullah A., Fan H., Jamil M.I., Khan F.U., Ullah R., Iqbal M., Ali A., Ullah B. (2021). Recent progress in silane coupling agent with its emerging applications. J. Polym. Environ..

[22] El-Batal A.I., Nasser H.A., Mosallam F.M. (2020). Fabrication and characterization of cobalt hyaluronic acid nanostructure via gamma irradiation for improving biomedical applications. Int. J. Biol. Macromol..

[23] Tapia-Guerrero Y.S., Del Prado-Audelo M.L., Borbolla-Jiménez F.V., Gomez D.M.G., García-Aguirre I., Colín-Castro C.A., Morales-González J.A., Leyva-Gómez G., Magaña J.J. (2020). Effect of UV and gamma irradiation sterilization processes in the properties of different polymeric nanoparticles for biomedical applications. Materials.

[24] da Silva Aquino K.A. (2012). Sterilization by gamma irradiation. Gamma Radiat..

[25] Ganguly S., Das P., Srinivasan S., Rajabzadeh A.R., Tang X.S., Margel S. (2024). Superparamagnetic amine-functionalized maghemite nanoparticles as a thixotropy promoter for hydrogels and magnetic field-driven diffusion-controlled drug release. ACS Appl. Nano Mater..

[26] Sun X., Yao F., Li J. (2020). Nanocomposite hydrogel-based strain and pressure sensors: A review. J. Mater. Chem. A.

[27] Wan X., Liu M., Zhang F., Xu L., Wang S. (2025). Interfacial Chemistry in Functional Hydrogel Coatings. Angew. Chem. Int. Ed..

[28] Wang X.-Q., Xie A.-Q., Cao P., Yang J., Ong W.L., Zhang K.-Q., Ho G.W. (2024). Structuring and shaping of mechanically robust and functional hydrogels toward wearable and implantable applications. Adv. Mater..

[29] Li M., Fan Y., Ran M., Chen H., Han J., Zhai J., Wang Z., Ning C., Shi Z., Yu P. (2024). Hydrogel coatings of implants for pathological bone repair. Adv. Healthc. Mater..

[30] Periyasamy T., Asrafali S.P., Lee J. (2025). Recent Advances in Functional Biopolymer Films with Antimicrobial and Antioxidant Properties for Enhanced Food Packaging. Polymers.

[31] Shah Y.A., Bhatia S., Al-Harrasi A., Khan T.S. (2024). Advancements in the biopolymer films for food packaging applications: A short review. Biotechnol. Sustain. Mater..

[32] Upadhyay P., Zubair M., Roopesh M.S., Ullah A. (2024). An overview of advanced antimicrobial food packaging: Emphasizing antimicrobial agents and polymer-based films. Polymers.

[33] Das P., Sherazee M., Marvi P.K., Ahmed S.R., Gedanken A., Srinivasan S., Rajabzadeh A.R. (2023). Waste-Derived Sustainable Fluorescent Nanocarbon-Coated Breathable Functional Fabric for Antioxidant and Antimicrobial Applications. ACS Appl. Mater. Interfaces.

[34] Saravanan A., Maruthapandi M., Das P., Ganguly S., Margel S., Luong J.H.T., Gedanken A. (2020). Applications of N-doped carbon dots as antimicrobial agents, antibiotic carriers, and selective fluorescent probes for nitro explosives. ACS Appl. Bio Mater..

[35] Das P., Ganguly S., Marvi P.K., Sherazee M., Tang X., Srinivasan S., Rajabzadeh A.R. (2024). Carbon Dots Infused 3D Printed Cephalopod Mimetic Bactericidal and Antioxidant Hydrogel for Uniaxial Mechano-Fluorescent Tactile Sensor. Adv. Mater..

[36] Das P., Ganguly S., Saha A., Noked M., Margel S., Gedanken A. (2021). Carbon-dots-initiated photopolymerization: An in situ synthetic approach for MXene/poly (norepinephrine)/copper hybrid and its application for mitigating water pollution. ACS Appl. Mater. Interfaces.

[37] Atchudan R., Edison T.N.J.I., Chakradhar D., Perumal S., Shim J.-J., Lee Y.R. (2017). Facile green synthesis of nitrogen-doped carbon dots using Chionanthus retusus fruit extract and investigation of their suitability for metal ion sensing and biological applications. Sens. Actuators B Chem..

[38] Qi H., Teng M., Liu M., Liu S., Li J., Yu H., Teng C., Huang Z., Liu H., Shao Q. (2019). Biomass-derived nitrogen-doped carbon quantum dots: Highly selective fluorescent probe for detecting Fe^3+^ ions and tetracyclines. J. Colloid Interface Sci..

[39] Shi J., Ni G., Tu J., Jin X., Peng J. (2017). Green synthesis of fluorescent carbon dots for sensitive detection of Fe^2+^ and hydrogen peroxide. J. Nanoparticle Res..

[40] Astafiev A.A., Shakhov A.M., Kritchenkov A.S., Khrustalev V.N., Shepel D.V., Nadtochenko V.A., Tskhovrebov A.G. (2021). Femtosecond laser synthesis of nitrogen-doped luminescent carbon dots from acetonitrile. Dye. Pigment..

[41] Samadi S., Jaymand M., Rahimpour F. (2025). Bioinspired electroconductive and antibacterial nanocomposite hydrogel composed of hyaluronic acid, aniline tetramer and clay as a scaffold for bone tissue engineering application. Int. J. Biol. Macromol..

[42] Ganguly S., Mondal S., Das P., Bhawal P., Das T.K., Ghosh S., Remanan S., Das N.C. (2019). An insight into the physico-mechanical signatures of silylated graphene oxide in poly (ethylene methyl acrylate) copolymeric thermoplastic matrix. Macromol. Res..

[43] Ghosh S.K., Das T.K., Ganguly S., Nath K., Paul S., Ganguly D., Das N.C. (2023). Silane functionalization of sodium montmorillonite and halloysite (HNT) nanoclays by ‘grafting to’method to improve physico-mechanical and barrier properties of LLDPE/clay nanocomposites. Polym. Bull..

[44] Zaca-Moran O., Díaz-Monge F., Rodríguez-Juárez A., Gómez-Muñoz C., Zaca-Moran P., Secundino-Sánchez O., Díaz-Reyes J. (2024). Synthesis and characterization of fluorescent carbon dots obtained from citrus x sinensis by an eco-friendly method. Results Chem..

[45] Das P., Bose M., Das A.K., Banerjee S., Das N.C. (2018). One-step synthesis of fluorescent carbon dots for bio-labeling assay. Macromolecular Symposia.

[46] Mewada A., Pandey S., Shinde S., Mishra N., Oza G., Thakur M., Sharon M., Sharon M. (2013). Green synthesis of biocompatible carbon dots using aqueous extract of Trapa bispinosa peel. Mater. Sci. Eng. C.

[47] Ganguly S., Das P., Itzhaki E., Hadad E., Gedanken A., Margel S. (2020). Microwave-synthesized polysaccharide-derived carbon dots as therapeutic cargoes and toughening agents for elastomeric gels. ACS Appl. Mater. Interfaces.

[48] Kanungo S., Gupta N., Rawat R., Jain B., Solanki A., Panday A., Das P., Ganguly S. (2023). Doped carbon quantum dots reinforced hydrogels for sustained delivery of molecular cargo. J. Funct. Biomater..

[49] Konwar A., Gogoi N., Majumdar G., Chowdhury D. (2015). Green chitosan–carbon dots nanocomposite hydrogel film with superior properties. Carbohydr. Polym..

[50] Das P., Ganguly S., Saravanan A., Margel S., Gedanken A., Srinivasan S., Rajabzadeh A.R. (2022). Naturally derived carbon dots in situ confined self-healing and breathable hydrogel monolith for anomalous diffusion-driven phytomedicine release. ACS Appl. Bio Mater..

[51] Wang Y.-Q., Xue Y.-N., Li S.-R., Zhang X.-H., Fei H.-X., Wu X.-G., Sang S.-B., Li X.-N., Wei M., Chen W.-Y. (2017). Nanocomposite carbon dots/PAM fluorescent hydrogels and their mechanical properties. J. Polym. Res..

[52] Wang Y., Xue Y., Wang J., Zhu Y., Zhu Y., Zhang X., Liao J., Li X., Wu X., Qin Y.-X. (2019). A composite hydrogel with high mechanical strength, fluorescence, and degradable behavior for bone tissue engineering. Polymers.

[53] Sachdev A., Matai I., Gopinath P. (2016). Carbon dots incorporated polymeric hydrogels as multifunctional platform for imaging and induction of apoptosis in lung cancer cells. Colloids Surf. B Biointerfaces.

[54] Singh S., Mishra A., Kumari R., Sinha K.K., Singh M.K., Das P. (2017). Carbon dots assisted formation of DNA hydrogel for sustained release of drug. Carbon.

[55] Kumar V., Vinayak A.K., Venkatachalam S., Muruganandam L. (2022). Moisture barrier behavior of polymer films on food packaging plastic materials. Int. J. Technol. Res. Eng..

[56] Ellingford C., Samantaray P.K., Farris S., McNally T., Tan B., Sun Z., Huang W., Ji Y., Wan C. (2022). Reactive extrusion of biodegradable PGA/PBAT blends to enhance flexibility and gas barrier properties. J. Appl. Polym. Sci..

[57] Lavrič G., Oberlintner A., Filipova I., Novak U., Likozar B., Vrabič-Brodnjak U. (2021). Functional nanocellulose, alginate and chitosan nanocomposites designed as active film packaging materials. Polymers.

[58] Long J., Zhang W., Zhao M., Ruan C.-Q. (2023). The reduce of water vapor permeability of polysaccharide-based films in food packaging: A comprehensive review. Carbohydr. Polym..

[59] Shi H., Jiang X., Liu G., Ma B., Lv Y., Xu P., Ma P., Zhang X., Liu T. (2024). Enhancement of PLA crystallization, transparency, and strength by adding the long aliphatic chains grafted CNC. Int. J. Biol. Macromol..

[60] Chen T., Li J., Xu J., Gao Y., Zhu S., Wang B., Ying G. (2023). Effect of acetylation of two cellulose nanocrystal polymorphs on processibility and physical properties of polylactide/cellulose nanocrystal composite film. Molecules.

[61] Younus M.M., Naguib H.M., Fekry M., Elsawy M.A. (2023). Pushing the limits of PLA by exploring the power of MWCNTs in enhancing thermal, mechanical properties, and weathering resistance. Sci. Rep..

[62] Alzahrani A. (2024). Fluorescent carbon dots in situ polymerized biodegradable semi-interpenetrating tough hydrogel films with antioxidant and antibacterial activity for applications in food industry. Food Chem..

[63] Althawab S.A., Alsulami T., Alzahrani H., Alzahrani A. (2024). Doped carbon dots reinforced flexible nanocomposite active polymer film for antibacterial and antioxidant activities. Colloids Surf. A Physicochem. Eng. Asp..

[64] Nille O.S., Patil A.S., Vibhute A.A., Shendage S.S., Tiwari A.P., Anbhule P.V., Sohn D., Gore A.H., Kolekar G.B. (2024). Route-dependent tailoring of carbon dot release in alginate hydrogel beads (HB-Alg@ WTR-CDs): A versatile platform for biomedical applications. Int. J. Biol. Macromol..

[65] Shao G., Wang S., Zhao H., Zhao G., Yang L., Zhu L., Liu H. (2022). Tunable arrangement of hydrogel and cyclodextrin-based metal organic frameworks suitable for drug encapsulation and release. Carbohydr. Polym..

[66] Xiao H., Verboven P., Tong S., Pedersen O., Nicolaï B. (2024). Hypoxia in tomato (*Solanum lycopersicum*) fruit during ripening: Biophysical elucidation by a 3D reaction–diffusion model. Plant Physiol..

[67] Joseph M., Xiao H., Postelmans A., Hertog M., Verboven P., Nicolaï B., Saeys W. (2024). Efficient estimation of gas exchange and respiration kinetics in apple using pathlength-resolved GASMAS. Postharvest Biol. Technol..

[68] Gao Y., Li J., Wang S., Jia J., Wu F., Yu G. (2025). Global inland water greenhouse gas (GHG) geographical patterns and escape mechanisms under different water level. Water Res..

