# Supplementary material for: Sustainable Gamma-Crosslinked Hyaluronic Acid-Carbon Quantum Dots Films for Active Packaging and Extended Fruit Shelf Life
Source: Nanomaterials (Basel). 2026 Sep 10;16(18):1129. doi: 10.3390/nano16181129 (PMC13610438; doi:10.3390/nano16181129)
Supplement: Supplementary file 1 [file nanomaterials-16-01129-s001.zip › nanomaterials-4546565-supplementary.pdf]

Supporting Information

Sustainable Gamma-Crosslinked Hyaluronic Acid-Carbon Quantum Dots Films for Active Packaging and Extended Fruit Shelf Life

Abdulhakeem Alzahrani * and Suleiman A. Althawab

Department of Food Science & Nutrition, College of Food and Agricultural Sciences, King Saud University, Riyadh 11451, Saudi Arabia

* Correspondence: aabdulhakeem@ksu.edu.sa

Characterizations

The chemical structure and functional groups of the synthesized BCDs and HT hydrogel films were analyzed using Fourier Transform Infrared (FTIR) spectroscopy on a Bruker Tensor 27 spectrometer in the range of 4000-400 cm^{-1}. The optical properties and photoluminescence behavior of the BCDs and nanocomposite films were investigated using a Cary 5000 UV–Vis–NIR spectrophotometer and a Horiba Fluorolog-3 spectrofluorometer, respectively. The surface morphology and microstructure of the films were examined using field emission scanning electron microscopy (FESEM, JEOL JSM-7610F). Mechanical properties, including tensile strength and elongation at break, were measured using a Universal Testing Machine (Instron 5943) at a crosshead speed of 5 mm min^{-1} according to ASTM D882. The water contact angle was recorded using a Krüss DSA100 drop shape analyzer to assess film wettability and surface hydrophilicity. Thermal stability of the films was determined using thermogravimetric analysis (TGA, TA Instruments Q500) under nitrogen atmosphere at a heating rate of 10 °C min^{-1}. The antioxidant activity of the films was analyzed via DPPH and ABTS radical scavenging assay using a UV–Vis spectrophotometer (Shimadzu UV-2600). In vitro cytotoxicity was evaluated using the MTT assay on 3T3 fibroblast cells with absorbance measured on a BioTek Synergy H1 microplate reader. Finally, barrier properties such as water vapor permeability (WVP) and oxygen transmission rate (OTR) were assessed using a MOCON Permatran-W 3/33 and MOCON OX-TRAN 2/21, respectively, following standard ASTM protocols.

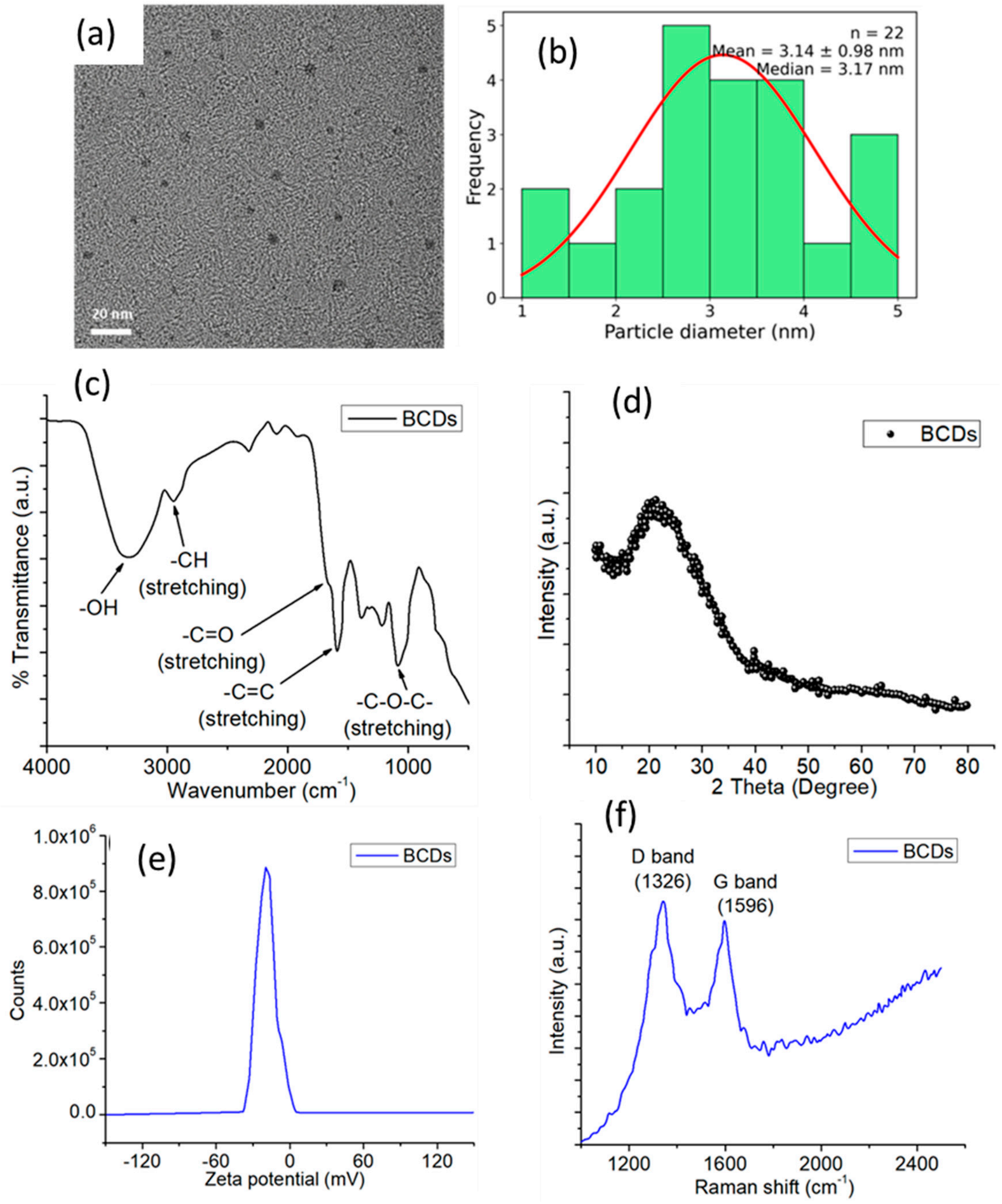

Figure S1 (a) TEM micrograph showing the morphology and dispersion of the synthesized BCDs; and (b) particle size distribution derived from TEM measurements, revealing an average particle

size of 3.14 ± 0.98 nm with a median size of 3.17 nm. (c) FTIR spectrum of BCDs showing surface functionalities of the carbon dots (d) XRD of BCDs (e) surface charge of the BCDs in aqueous medium (f) Raman spectrum of BCDs after dried in silicon wafer

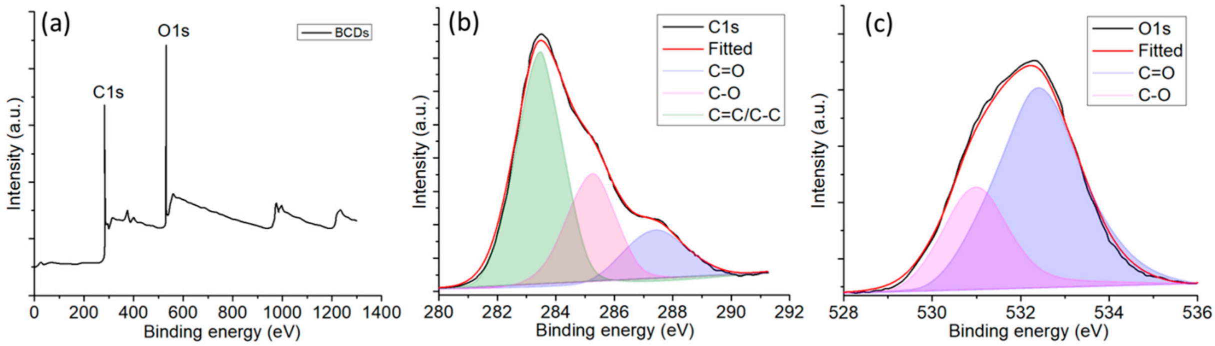

Figure S2 (a) XPS surface spectrum of BCDs (b) high resolution C1s spectrum of BCDs (c) high resolution O1s spectrum of BCDs

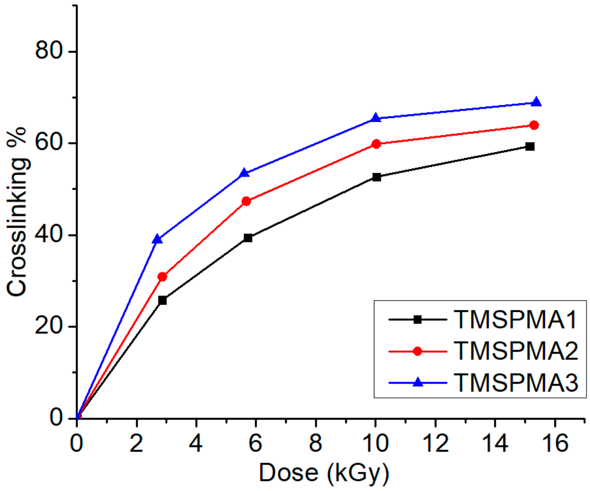

Figure S3 Crosslinking percentage of the hybrid film in function of dose and TMSPMA (monomer) concentration.

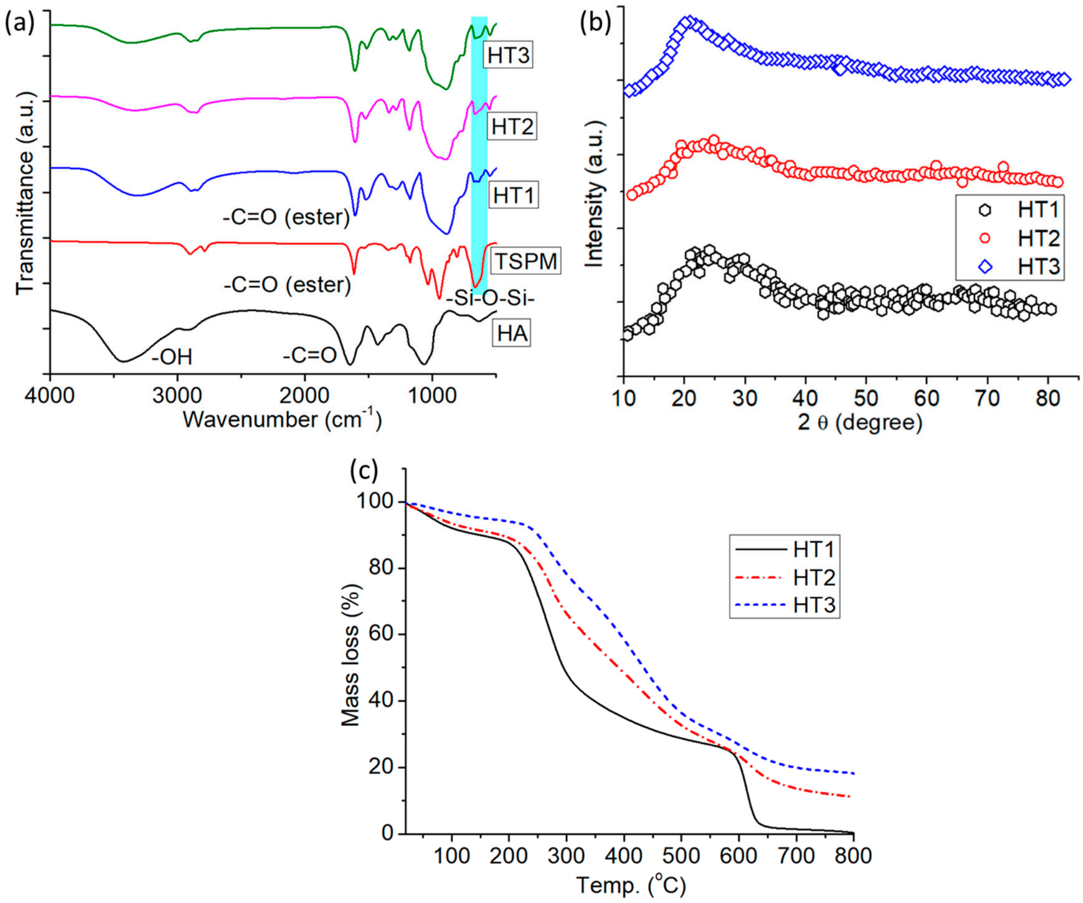

Figure S4 (a) FTIR spectra of pristine hyaluronic acid (HA), monomer (TSPM), and the composite films (b) XRD of CDs loaded nanocomposite hydrogels (c) TGA thermograms of the cured nanocomposite films

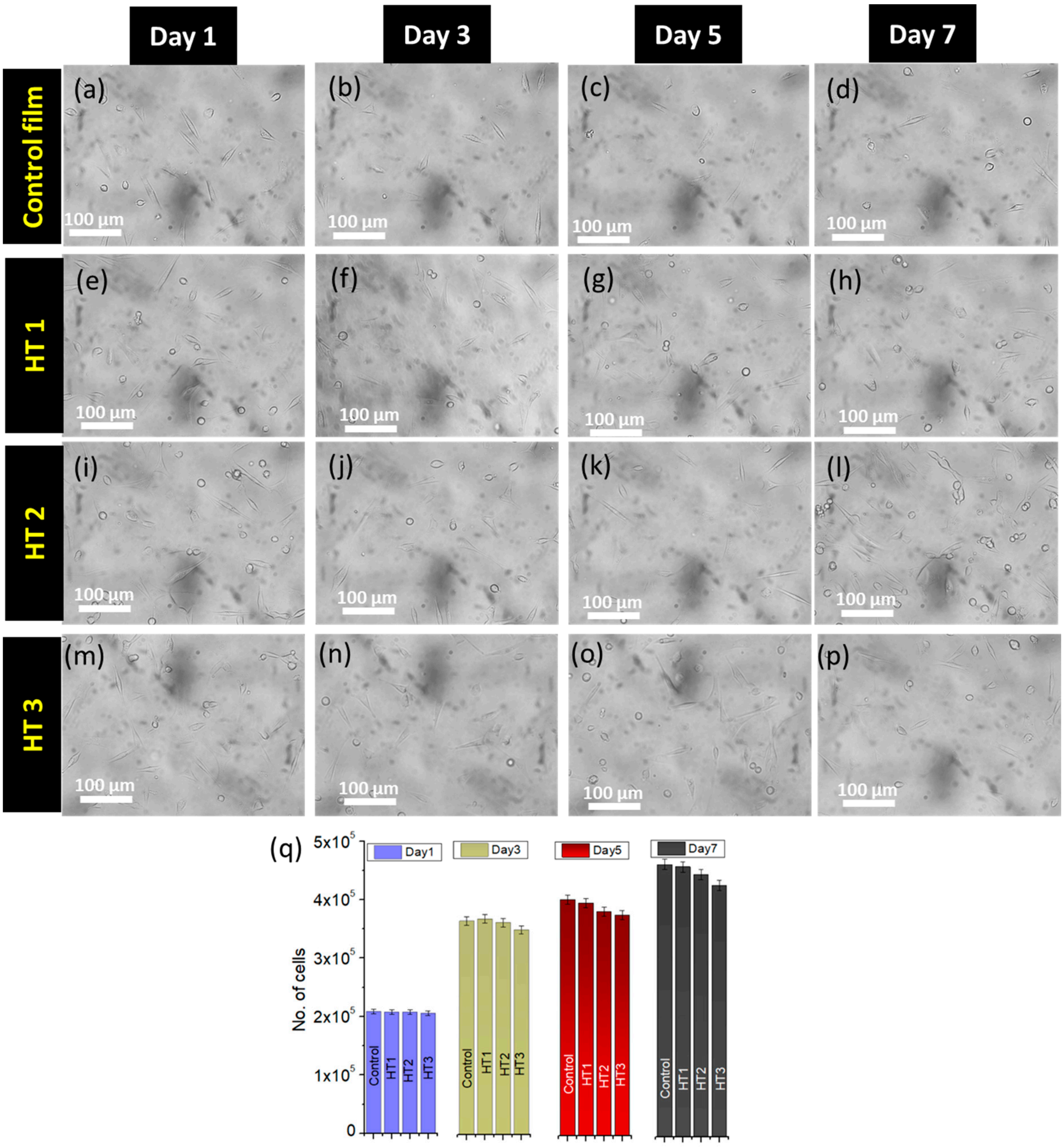

Figure S5 (a-p) Cell proliferation studies of the control and nanocomposite hydrogels showing cell growth and achieving their morphology (q) MTT test of the nanocomposite hydrogel films from Day 1-7.

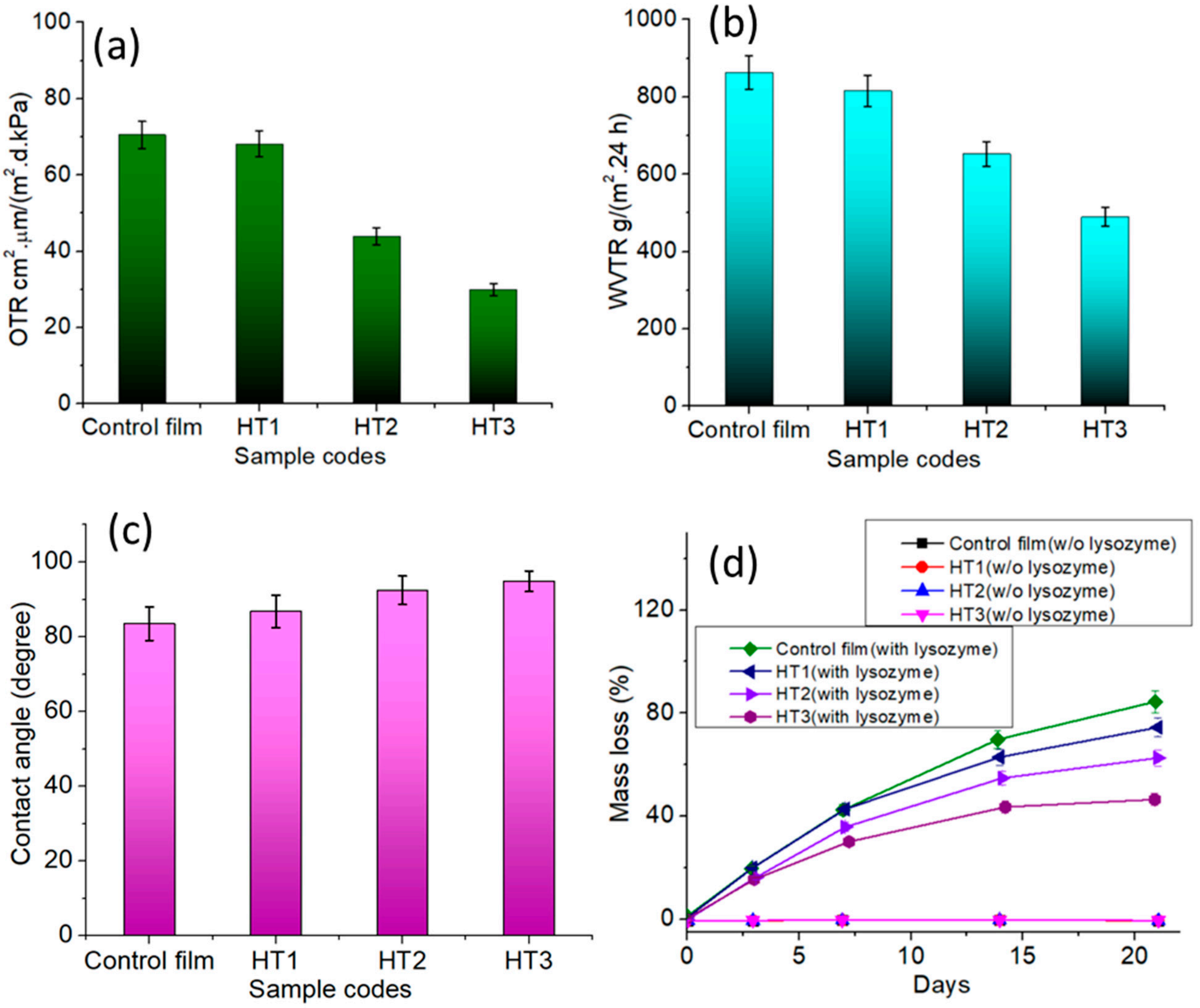

Figure S6 (a) Oxygen transmission rate (OTR) of different films (b) water valor transmission rate (WVTR) of different films (c) water contact angle of pristine film and effect of CDs on their wetting behavior (d) biodegradability test of the prepared films showing how CDs affect their biodegradability
